# Bayesian copy number detection and association in large-scale studies

**DOI:** 10.1186/s12885-020-07304-3

**Published:** 2020-09-07

**Authors:** Stephen Cristiano, David McKean, Jacob Carey, Paige Bracci, Paul Brennan, Michael Chou, Mengmeng Du, Steven Gallinger, Michael G. Goggins, Manal M. Hassan, Rayjean J. Hung, Robert C. Kurtz, Donghui Li, Lingeng Lu, Rachel Neale, Sara Olson, Gloria Petersen, Kari G. Rabe, Jack Fu, Harvey Risch, Gary L. Rosner, Ingo Ruczinski, Alison P. Klein, Robert B. Scharpf

**Affiliations:** 1 Department of Biostatistics, Johns Hopkins Bloomberg School of Public Health, Baltimore, MD USA; 2 Department of Oncology The Sidney Kimmel Comprehensive Cancer Center, Johns Hopkins University School of Medicine, Baltimore, MD USA; 3 Department of Epidemiology and Biostatistics, University of California, San Francisco, San Francisco, CA USA; 4 Genetics Section, International Agency for Research on Cancer, Lyon, France; 5 Department of Epidemiology, Johns Hopkins Bloomberg School of Public Health, Baltimore, MD USA; 6 Department of Epidemiology and Biostatistics, Memorial Sloan Kettering Cancer Center, New York, 10065 NY USA; 7 Lunenfeld-Tanenbaum Research Institute of Mount Sinai Hospital, Toronto, M5G 1x5 Ontario Canada; 8 Department of Medicine, Johns Hopkins University School of Medicine, Baltimore, MD USA; 9 Department of Pathology, Sol Goldman Pancreatic Cancer Research Center, Johns Hopkins School of Medicine, Baltimore, MD USA; 10 Department of Epidemiology, Cancer Prevention & Population Sciences, UT MD Anderson Cancer Center, Houston, 77030 TX USA; 11 Department of Gastroenterology, Hepatology, and Nutrition Service, Memorial Sloan Kettering Cancer Center, New York, 10065 NY USA; 12 Department of Gastrointestinal Medical Oncology, University of Texas MD Anderson Cancer Center, Houston, 77030 TX USA; 13 Department of Chronic Disease Epidemiology, Yale School of Public Health, Yale Cancer Center, New Haven, CT USA; 14 Population Health Department, QIMR Berghofer Medical Research Institute, Brisbane, 4029 Australia; 15 Department of Health Sciences Research, Mayo Clinic College of Medicine, Rochester, 55905 MN USA

**Keywords:** Pancreatic cancer, SNP array, Copy number variants, Genome-wide association, CNPBayes, Batch effects

## Abstract

**Background:**

Germline copy number variants (CNVs) increase risk for many diseases, yet detection of CNVs and quantifying their contribution to disease risk in large-scale studies is challenging due to biological and technical sources of heterogeneity that vary across the genome within and between samples.

**Methods:**

We developed an approach called CNPBayes to identify latent batch effects in genome-wide association studies involving copy number, to provide probabilistic estimates of integer copy number across the estimated batches, and to fully integrate the copy number uncertainty in the association model for disease.

**Results:**

Applying a hidden Markov model (HMM) to identify CNVs in a large multi-site Pancreatic Cancer Case Control study (PanC4) of 7598 participants, we found CNV inference was highly sensitive to technical noise that varied appreciably among participants. Applying CNPBayes to this dataset, we found that the major sources of technical variation were linked to sample processing by the centralized laboratory and not the individual study sites. Modeling the latent batch effects at each CNV region hierarchically, we developed probabilistic estimates of copy number that were directly incorporated in a Bayesian regression model for pancreatic cancer risk. Candidate associations aided by this approach include deletions of 8q24 near regulatory elements of the tumor oncogene *MYC* and of Tumor Suppressor Candidate 3 (*TUSC3*).

**Conclusions:**

Laboratory effects may not account for the major sources of technical variation in genome-wide association studies. This study provides a robust Bayesian inferential framework for identifying latent batch effects, estimating copy number, and evaluating the role of copy number in heritable diseases.

## Background

Germline copy number variants (CNVs) can be identified from hybridization-based arrays and capture-based sequencing with measures of abundance derived from intensities and normalized read depth, respectively. Biological and technical sources of heterogeneity of these measurements are intricately related. For example, the GC composition of genomic DNA effects PCR efficiency and leads to autocorrelated measures of DNA abundance across the genome [1–4]. These effects have been shown to be heterogeneous across the genome and to differ in both magnitude and direction between samples [1, 5, 6]. Hidden Markov models and nonparametric segmentation algorithms for CNV detection over-segment low-quality data where these effects are the most pronounced, contributing to false positive deletion and duplication calls.

For studies with hundreds to thousands of samples, estimation of copy number at regions known to harbor CNVs has the potential to improve sensitivity and specificity as technical sources of variation across the genome are largely controlled when limited to a focal genomic region (less than 1 MB) and variation between samples can be explicitly modeled [7–12]. Such CNV regions are of particular interest for a comprehensive assessment of common genetic variants and their relationship to disease. However, scaling marginal models to CNV regions across the genome and to thousands of samples has proved challenging. The sources of technical variation giving rise to batch effects are typically unknown. Standard approaches for estimating latent batch effects in high-throughput experiments such as surrogate variable analysis are not appropriate when the biological variation of interest (copy number) is also unknown [13]. In addition, the statistical framework for copy number estimation must flexibly accommodate deletions and duplications of varying size and allele frequencies. Symptomatic of the challenges in copy number analyses and the limitations of current methods, genome-wide association studies rarely incorporate copy number in the initial publication despite their well established role in neurodevelopmental disorders [14–16] and cancer [17]. Previous genome-wide studies of pancreatic cancer and copy number have been limited in size with fewer than 250 pancreatic cancer patients [18, 19].

Here, we performed genome-wide copy number analysis for 3,974 cases and 3,624 controls in PanC4 using Illumina’s OmniExpress Exome array. We developed methods for identifying latent batch effects at CNV regions from commonly available experimental data on the samples. The effects of copy number and batch on measures of DNA abundance were modeled hierarchically through implementation of Bayesian finite mixture models. For the association model, we used Markov Chain Monte Carlo (MCMC) to incorporate the uncertainty of the integer copy numbers in a logistic regression model of pancreatic cancer risk.

## Methods

*The Pancreatic Cancer Case and Control Consortium*

Clinical and demographic characteristics of the cases and controls in PanC4 and recruitment methods have been previously described [20]. All samples were processed using GenomeStudio (version 2011.1, Genotyping Module 1.9.4). For GC-correction, we sampled a random subset of 30,000 Illumina probes, fit LOESS with span 1/3 to the scatterplot of log2*R* ratios and probe GC content, and predicted the log2*R* ratios for the full probeset from the LOESS model. For spatial correction, we applied LOESS to the GC-corrected log2*R* ratios at single nucleotide polymorphisms (SNPs) with balanced allele frequencies (0.4<B allele frequency<0.6) ordered by genomic position within each chromosome arm and predicted the GC-corrected log2*R* ratios for the full probeset, including SNPs with imbalanced allele frequencies. The residuals from the spatial LOESS were used in all downstream analyses with CNPBayes.

*CNV regions:*

CNV regions identified for further analysis by CNPBayes were obtained from the collection of CNVs identified from a hidden Markov model as well as known CNV regions from the 1000 Genomes Project. For the former, we fit a 5-state hidden Markov model implemented in the R package VanillaICE (version 1.40.0) using default parameter settings [21]. To obtain a high confidence call set, we removed CNVs with fewer than 10 probes, CNVs with posterior probability less than 0.9, and restricted inference to autosomal chromosomes. To assess the effect of spatial adjustment on copy number inference, we stratified the samples into deciles of median absolute deviation and autocorrelation coefficient (ACF) and compared the results of the 5-state HMM fit after GC-correction to the CNVs identified after spatial correction. Concordance of CNVs identified by the HMMs was defined by ≥ 50% reciprocal overlap [22].

CNV regions were defined by the set of non-overlapping disjoint intervals across the pooled set of all CNVs from cases and controls. We computed the number of subjects with a CNV overlapping each disjoint interval, retaining intervals where CNVs were identified in at least 150 participants. Among the disjoint intervals, we defined the CNV region as the minimum start and maximum end for which at least 50 percent of the copy number altered samples had a CNV. For CNV regions obtained from the 1000 Genomes Project, we excluded regions that did not span at least 4 markers on the OmniExpress array.

*Batch effects:*

We evaluated both chemistry plate and DNA extraction method as surrogates for batch effects. With provisionally defined batches by plate or extraction method, we compared the empirical cummulative distribution function (eCDF) of the mean log2*R* ratio between two batches (excluding samples with log2*R* ratio <−1) by the Kolmogorov-Smirnov (K-S) test statistic. For two batches without a statistically significant difference in the K-S statistic at a type 1 error of 0.01, the batches were combined into a single new batch. This procedure was applied recursively at each CNV region until no further grouping of batch surrogates could be obtained.

*Hierarchical Bayesian mixture model:*

Hierarchical Bayesian mixtures of *t*-distributions were used to cluster median log2*R* ratios within a CNV region. Let *r*_*ib*_ and *z*_*ib*_ denote the observed one-dimensional summary of log2 ratios measured from the array and the true (but latent) mixture component, respectively, for the *i*th individual in batch *b*. Given *z*_*ib*_ is some integer *h* (*h*∈{1,…,*K*} for a *K*-component model), our sampling model for the observed data is a shifted and scaled *t*-distribution with *d* degrees of freedom, mean *θ*_*hb*_, and standard deviation *σ*_*hb*_ that depends on batch:

$$\begin{aligned} \left[r_{ib} | z_{ib}=h,\theta_{hb}, \sigma^{2}_{hb}, U_{ib}\right] &\sim \text{Normal}\left(\theta_{hb}, \frac{\sigma_{hb}^{2}}{U_{ib}/d}\right), \\ z_{ib} | \pi_{b1}, \ldots, \pi_{bK} &\sim \text{Multinomial}(\pi_{b1}, \ldots, \pi_{bK}), \\ \pi_{b1}, \ldots, \pi_{bK}| \alpha^{\pi}_{1}, \ldots, \alpha^{\pi}_{K} &\sim \text{Dirichlet}\left(\alpha^{\pi}_{1}, \ldots, \alpha^{\pi}_{K}\right), \text{~and}\\ U_{ib} | d &\sim \text{Gamma}(d/2, d/2). \end{aligned} $$

The degrees of freedom *d* controls robustness to outliers with larger values approximating a mixture of normal distributions [23]. To stabilize the mean and precision of batches having fewer samples, we model these parameters hierarchically with computationally convenient conjugate priors. Our sampling model for the batch means is normal and the precisions are Gamma,

$$\begin{aligned} \theta_{hb} | \mu_{h}, \tau_{h}^{2} &\sim \text{Normal}\left(\mu_{h}, \tau_{h}^{2}\right) \text{~and~} \\ {\tilde \sigma}_{hb}^{2} | \nu_{0}, \sigma_{0}^{2} &\sim \text{Gamma}\left(\frac12 \nu_{0}, \frac12 \nu_{0}\sigma_{0}^{2}\right), \end{aligned} $$

with *μ*_*h*_ representing the overall mean for component *h*, *τ*_*h*_ capturing the heterogeneity of the batch-specific means, and ${\tilde \sigma }_{hb}^{2} = 1/\sigma ^{2}_{hb}$. Conjugate priors on $\mu _{h}, ~\tau _{h}^{2}, ~\sigma _{0}$, and *ν*_0_ are given by

$$\begin{aligned} \mu_{h} | \mu_{o}, \tau_{0}^{2} &\sim \text{Normal}(\mu_{0}, \tau_{0}^{2}) \text{~~for~} h=1, \ldots, K,\\ {\tilde \tau}_{h}^{2} &\sim \text{Gamma}\left(\frac12 \eta_{0}, \frac12 \eta_{0} m_{0}^{2} \right), \\ \sigma_{0}^{2} | a_{0}, b_{0} &\sim \text{Gamma}(a_{0}, b_{0}), \text{~and}\\ \nu_{0} | \beta &\sim \text{Geometric}(\beta).\\ \end{aligned} $$

Label switching is well known in Bayesian mixture models. In addition to visual inspection of the chains, we compared the ordering of parameter means for subsequences of the chains. Label switching occurred most often when the number of components specified was too large and these models were discarded. In addition, we use an informative prior on $\tau ^{2}_{h}$ that governs the heterogeneity of the mean for mixture component *h* across the batches (Table S3). This prior discourages label switching at bona fide copy number polymorphisms since this would typically result in a large variance of the component means.

As all priors were conjugate, we used Gibbs sampling to approximate the joint posterior distribution of $p\left (\pmb {\mu }, \pmb {\tau }, \pmb {\theta }, \pmb {\sigma }^{2}, \pmb {z}, \pmb {\pi }, \nu _{0}, \tau _{0}^{2}, \sigma _{0}^{2}, m_{0}^{2}, \eta _{0} | \pmb {r}, K, d\right)$. We refer to the above implementation of the Gibbs sampler with batch-specific means and variances as the multi-batch (MB) model. CNPBayes provides several more parsimonious alternatives to the MB model, including a pooled variance model (MBP) with a single variance estimate per batch. In addition, we evaluated models with a single batch (SB) and a single batch model with pooled variances (SBP) that are special cases of the MB and MBP models, respectively. Hyper-parameters used in the MB, MBP, SB, and SBP models were the defaults in version 1.11.2 of CNPBayes (Table S3).

*Implementation:*

Heavy-tailed marginal distributions of the one-dimensional log2*R* ratio summaries were often a consequence of batch effects. When latent batch effects were estimated as previously described, near-Gaussian mixtures were needed to fit the very peaked densities of log ratios near the central mode. As residual outliers and lack of normality after taking batch effects into account were often asymmetric and could be captured by an additional mixture component, we fit finite mixtures of near-Gaussian distributions with *d*=100 degrees of freedom in both the PanC4 application and simulations. Estimation of *d*, for example from a discrete uniform prior ([24, 25]), is not currently available in CNPBayes.

For studies of germline CNVs, extreme observations in the left-tail typically correspond to homozygous deletions and, when rare, may be present in a subset of the estimated batches. The consequences of a rare deletion present in a subset of batches are two-fold: (1) due to the hierarchical nature of the model, a mixture-component with a very large variance will be needed to accommodate the extreme observations and (2) the mixture component indices may correspond to different copy number states between batches, complicating subsequent efforts to map mixture component indices to integer copy numbers. Rather than exclude these observations, we augment the observed data with simulated homozygous deletions. The simulated observations ensure the mixture component indices capture the same latent copy number in each batch. We rationalize this approach as being comparable to an empirically derived prior that large negative values at such germline CNV regions are not outliers of the hemizygous and diploid states but bona fide homozygous deletions. Since our model does not assume a one-to-one mapping between mixture components and copy number nor that any of the alterations identified will be in Hardy Weinberg equilibrium (HWE), the assessment of HWE for germline CNVs can be a useful post-hoc quality control. While evidence against HWE does not necessarily indicate problems with the CNV calling, support for HWE would be unlikely if there were major sources of technical variation not yet accounted for.

As fitting hierarchical Bayesian mixture models is computationally intensive, we implemented ad hoc procedures to reduce computation (see also Scalability and Software). First, we considered only 3 and 4 component models when homozygous deletions were apparent (one- and two-component models were not evaluated). Secondly, MB and MBP models were only evaluated when more than 2% of the samples had a posterior probability < 0.99 in the SB and/or SBP models. Thirdly, for each model under consideration, we independently initialized 10 models with parameters randomly sampled from their priors and ran a short burnin of 200 iterations for each model. Only the model with the largest log likelihood was selected for an additional 500 burnin simulations and 1000 simulations post-burnin. Finally, to aid comparison between hierarchical SB, SBP, MB, and MBP models, the CNPBayes package implements Chib’s method to estimate the marginal likelihood [26], allowing estimates of the relative evidence between two models by Bayes factors. However, as estimation of the marginal likelihoods requires additional MCMC simulations, we only computed marginal likelihoods when the difference of simple post-hoc statistical summaries, such as the log likelihood evaluated at the last iteration, was small (e.g., <10). CNPBayes automatically provides posterior predictive distributions of the CNV region summaries for goodness of fit assessments, allowing simple verification that the selected model is not simply the best of many poor fitting models. We recommend running multiple chains to assess convergence [27] and additional MCMC simulations with an increased thin parameter when autocorrelation is substantial.

*Genotyping mixture components:*

For genotyping the mixture components at a CNP region, our goal was to identify the set of integer copy numbers that would most likely give rise to the observed B allele frequencies (BAFs) at SNPs in this genomic region. We excluded samples that were not assigned to a single mixture component with high posterior probability since these would be less informative. Denoting the mapping of mixture component indices *h* to integer copy numbers by *f*(*h*), the likelihood across SNPs indexed by *j* and samples indexed by *i* conditional on the mapping is

$$\begin{aligned} p(\pmb{b} | f(\pmb{h}), \pmb{\psi}) &= \prod_{i} \prod_{j} p(b_{ij} | f(h_{i}), \pmb{\psi}), \\ p(b_{ij} | f(h_{i}), \pmb{\psi}) &= \sum_{g \in G} p(b_{ij} | \pmb{\psi}_{g}) p(g | f(h_{i})), \\ p(b_{ij} | \pmb{\psi}_{g})&= \text{dbeta}(b_{ij} | \pmb{\psi}_{g}), \text{~and} \\ p(g | f(h_{i})) &= \text{dbinom}\left(g | p_{jB}, |G(f(h))|-1\right), \text{~where} \end{aligned} $$

dbeta and dbinom are shorthand for the densities for the beta and binomial distributions. For the binomial density, |·| denotes the cardinality of the set and *p*_*jB*_ the frequency of the B allele at SNP *j* in the population of PanC4 participants. The above likelihood averages over the set *G* of possible allele specific copy numbers ordered by the number of B alleles and indexed by *g* (e.g., *G*(2)∈{*AA*,*AB*,*BB*}; Table S4). Shape and scale parameters (*ψ*_*g*_) for the Beta distribution conditional on the allelic copy numbers are provided in Table S5. Evaluating one-to-one (e.g., *f*({1,2,3})→{0,1,2} for a deletion polymorphism) and many-to-one mappings (e.g., *f*({1,2,3})→{2,2,2}), we selected the mapping that maximized the above likelihood on the log-scale.

*Simulation:*

Affymetrix 6.0 data for 990 phase 3 HapMap samples processed on 16 chemistry plates were obtained from Wellcome Sanger Institute (https://www.sanger.ac.uk/resources/downloads/human/hapmap3.html) [28]. A region on chr4 70,122,981-70,231,746 containing 53 nonpolymorphic markers and 1 SNP spans a common copy number polymorphism. To establish a baseline for which both CNPBayes and CNVCALL correctly identify the copy number for all samples, we subtracted 3 from the log2*R* ratios for samples with apparent homozygous deletions. To simulate batch effects, we simulated a Bernoulli random variable with probability of success 0.5 for each of the 16 chemistry plates. For a plate *k* where the Bernoulli random deviate was 1, we rescaled the data by a factor *ξ* and shifted the means by a normal random deviate centered at *δ* such that the simulated log2*R* ratio (*r*^∗^) for marker *i* in sample *j* with integer copy number *c* becomes $r^{*}_{ijk} = (r_{ijk} - {\bar r}_{c})\times \xi + {\bar r}_{c} + \epsilon _{ijk}$, where *ε*_*ijk*_∼*N*(*δ*,0.02^2^) for values of *δ*∈{0,0.3,0.4,0.5} and *ξ*∈{1,1.25,1.50,1.75,2}. Applying CNVCALL to this data, the matrix of *r*^∗^ was summarized by the first principal component and mixture models with 3-5 components were evaluated using default parameters. As CNVCALL merges mixture components based on the extent of overlap of the component-specific densities but does not genotype the merged mixture components, we subtracted one from the merged mixture component indices. For CNPBayes, we explored SB, SBP, MB, and MBP models of 3 - 4 components with chemistry plate as the surrogate variable, median $\pmb {r}_{i}^{*}$ as one-dimensional summaries for each sample, and default values for hyperparameters. Mixture components were genotyped using the BAFs from the SNPs in this region as previously described.

*Bayesian logistic regression model for pancreatic cancer:*

For each CNP region, we modeled the case-control status *y*_*i*_ for individual *i* as:

$$\begin{array}{*{20}l} \left[y_{i} | \pmb{\gamma}, \pmb{X}_{i}, z, \beta, C_{i}\right] &\sim \text{Bernoulli}(\theta_{i}), \\ \text{logit}(\theta_{i}) =& \beta_{0} + \beta_{1}\text{age}_{i} + \beta_{2}\text{male}_{i} + \beta_{3}\text{PC1}_{i} \\&+ \beta_{4}\text{PC2}_{i} + \beta_{5}\text{PC3}_{i} + \beta_{6}I_{[\text{high quality}]}\\ &+ z \times \left(\beta_{7} C_{i} + \beta_{8} C_{i} * I_{[\text{high quality}]}\right)\\ \beta_{j} \sim& \text{Cauchy}(0, 2.5^{2}) ~~\text{for \(j=0, \ldots, 8\)},\\ z \sim& \text{Bernoulli}(0.5), \text{~and}\\ C_{i} \sim& \text{Multinomial}(\pi^{*}_{i1}, \ldots, \pi^{*}_{iG}),\text{~where}\\ \pi_{ig}^{*} =& \sum_{h: h \in \{f(h)=g\}} \pi_{ih}. \end{array} $$

All continuous independent variables were mean centered, including PC1, PC2, and PC3 denoting the first three principal components of the SNP genotype matrix in PanC4 [20]. An indicator for the collection of high quality samples for CNV analyses, *I*_[high quality]_, was defined as 1 for samples in this set and 0 otherwise. As the integer copy number *C*_*i*_ was not observed, we treated *C*_*i*_ as a parameter measured with error given by the aggregated posterior probabilities of the mixture component indices after genotyping, $\pi ^{*}_{ig}$. We used JAGS version 4.3.0 with a thin parameter of 25 and 5000 iterations to obtain posterior distributions of these parameters by MCMC [29].

*Scalability and software:*

Hierarchical mixture models were fit to a random sample of 1000 observations from the 7,598 available participants at each CNV region, and to all samples with apparent homozygous deletions. We parallelized our analysis so that all regions were evaluated simultaneously. Bayesian logistic regression models fit independently to each CNV region were also evaluated in parallel. CNPBayes is available from github (https://github.com/scristia/CNPBayes).

## Results

**Overview of study**

**Fig. 1:**
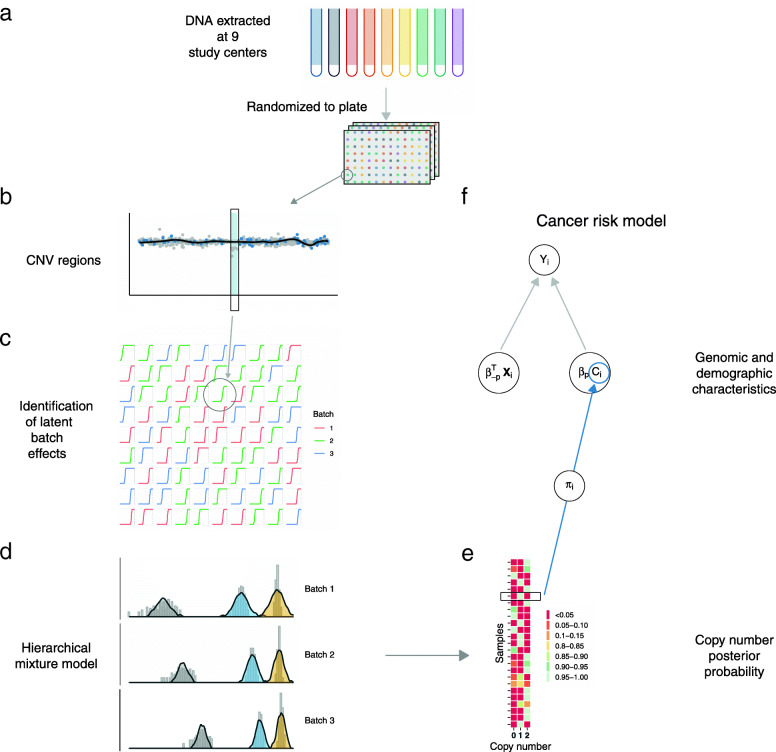
Overview of sample processing, estimation of batch effects and copy number, and risk model for pancreatic cancer. **a** DNA samples for pancreatic cancer cases and healthy controls were obtained from 9 different study centers and processed centrally where samples were randomized to chemistry plates. **b** Initial preprocessing of these samples identified candidate CNV regions. As the principal sources of batch effects were unknown, we developed an approach to identify latent batch effects by clustering empirical cummulative distribution functions (eCDFs) of CNV region summaries (**c**) and to genotype these samples via a Bayesian hierarchical mixture model (**d**). Uncertainty of the copy number genotypes (**e**) was propagated from the genomic analyses to the Bayesian logistic regression model for pancreatic cancer risk (**f**)

DNA specimens from 7598 European ancestry participants in this consortium were collected at 9 study sites using varying methods of DNA extraction [20]. Randomization of samples to chemistry plates, DNA amplification by PCR, and SNP genotyping using Illumina’s OmniExpress Exome-8 array were performed centrally at the Center for Inherited Disease Research (CIDR) (Fig. 1). CNV regions were extracted from the 1000 Genomes project [30] or identified from analysis of the PanC4 samples. Low-level copy number summaries were obtained for each participant by computing the median log2*R* ratios across available markers from the Illumina array spanned by the candidate CNV region. Independently for each region, we identified latent batch effects in the low-level summaries and fit a Bayesian hierarchical mixture model across the estimated batches using CNPBayes. To model the relationship between copy number and pancreatic cancer risk, we fit a Bayesian logistic regression model that included integer copy number as a covariate measured with error. The copy number measurement error for each participant was obtained from the posterior probabilities in the CNPBayes hierarchical model.

**Copy number analyses**

Log _2_*R* ratios for each participant were GC-corrected using loess. Measures of data quality following GC-correction include the median absolute deviation and lag-10 autocorrelation of autosomal log2*R* ratios ordered by genomic position. Data quality was high for the majority of PanC4 participants (Figure S1A), though approximately 11% of participants had autocorrelations greater than 0.1. To reduce the spatial autocorrelation, we developed a scatterplot smoother for the log2*R* ratios that was locally weighted by genomic position (Methods). Following the spatial correction, nearly all samples (≈ 98%) had low autocorrelation (Figure S1B). Rare and common CNVs identified by a 5-state HMM before and after spatial correction revealed near perfect concordance for samples in the first nine deciles of ACF (high quality samples) with sharply lower concordance among samples in the highest decile irrespective of CNV size (Figure S2). Hereafter, we refer to the set of 1,560 samples in the highest decile of ACF (ACF ≥ 0.06) as low quality samples and the remaining 6,038 samples in the first nine deciles (ACF < 0.06) as high quality samples.

To evaluate whether copy number inference could be improved by multi-sample methods that directly incorporate batch and other technical sources of variation between samples, we focused our analysis on 217 regions from the 1000 Genomes Project where CNVs were reported in at least 0.1% of European ancestry participants and that encompassed at least four probes on the Illumina OmniExome array (Table S1). Additionally, we identified 46 regions for which deletions or duplications were identified in at least 2% of the PanC4 participants by the HMM applied to the spatially corrected log2*R* ratios. Collectively, the 263 regions comprised 11.5 Mb of the coding genome and 6.4 Mb of the non-coding genome.

**Fig. 2:**
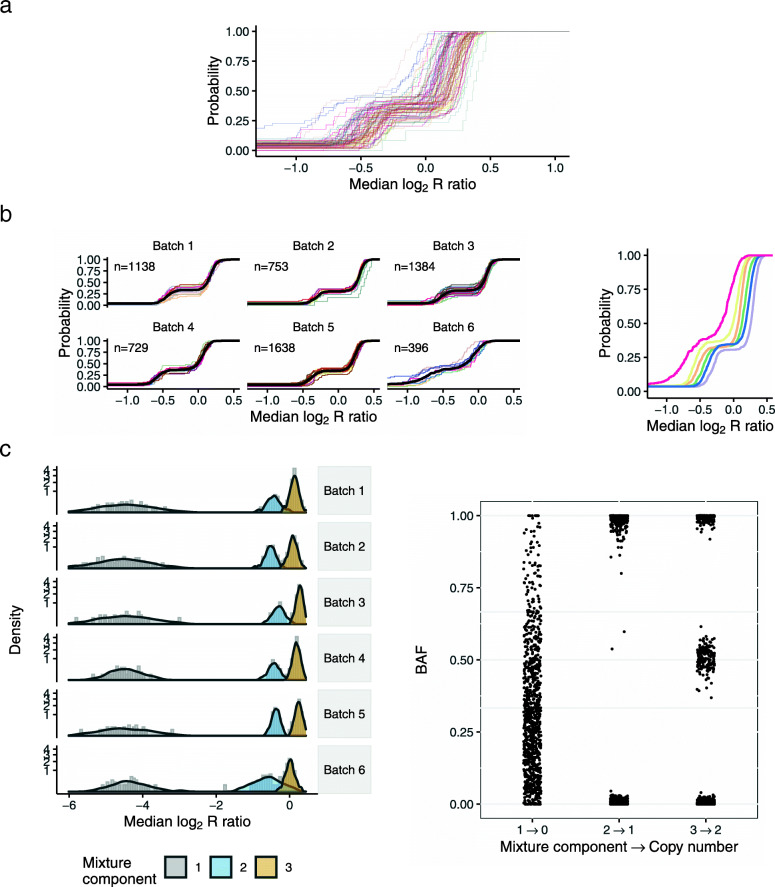
Identification of batch surrogates. **a** Plate-specific eCDFs of the average log2*R* ratio for a region on chr5 (155,475,886-155,488,649bp). **b** The plate-specific eCDFs were grouped by Kolmogorov-Smirnov test statistics, forming batches. The batch-specific eCDFs after grouping plates (right). The eCDFs between batches typically differed by a location shift, though here Batch 6 also captured samples with higher variance. **c** Single- and multi-batch mixture models were evaluated at each CNP. Densities from the posterior predictive distributions overlay the histograms of the 3-component multi-batch model (left). Adjusted for batch, only three components were needed to fit the apparent deletion polymorphism. B allele frequencies were used to genotype the mixture components. The mapping from the mixture component indices to copy number is indicated by the arrows on the x-axis labels (right)

**Fig. 3:**
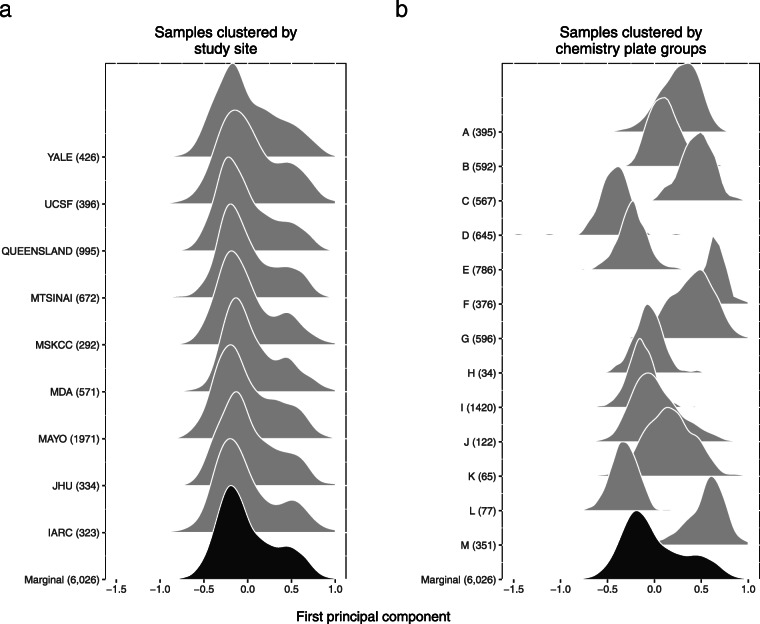
Study site does not capture the major sources of technical variation. Hybridization intensities were available for four probes in a CNP region on chr4 spanning 9,370,866 bp - 9,410,140 bp (CNP_051). Restricting our analysis to high quality samples, we used the first principal component (PC1) as a one-dimensional summary of the 4 x 6,026 matrix of log2*R* ratios. The density of the PC1 summaries marginally (black) and stratified by study site (gray) are bimodal, suggesting a copy number polymorphism **a**. However, stratification of the PC1 summaries by grouping chemistry plates with similar eCDFs reveals an obvious batch effect (**b**). For example, chemistry plates in group E comprised of 786 samples originating from all nine study sites has a markedly different distribution than the 567 samples processed on group C chemistry plates

Available multi-sample methods for modeling copy number assume the major sources of batch effects are known (e.g., laboratory or study site). Here, DNA samples were collected from multiple study sites and processed on 94 chemistry plates at a central lab. To identify batch surrogates for the central lab, we developed an approach for grouping chemistry plates with a similar median log2*R* ratio eCDF (Fig. 2a and b). As an example of these sources of heterogeneity at a single CNV region on chromosome 4, we summarized the log2*R* ratios for 6,026 high quality samples by the first principal component (PC1) and stratified the PC1 summaries by study site (Fig. 3a) or PCR batch surrogates (Fig. 3b). While the density of PC1 is bimodal when stratified by study site and consistent with a copy number polymorphism, stratification by the eCDF-derived batch surrogates revealed obvious batch effects (e.g., plate group C with 567 samples and plate group E with 786 samples; Fig. 3b). As PCR efficiency is known to be affected by GC content and can vary along the genome, we identified batch surrogates for each CNV region. The median number of batches across the 263 CNV regions was 4 with multiple batches identified for the majority of regions (Figure S3).

Our sampling model for the median log2*R* ratio is a mixture of *t* distributions with component-specific means and variances modeled hierarchically across batches (Fig. 2c). Following the probabilistic assignment of samples to mixture components, we genotyped the mixture components using the available BAFs at SNPs (Fig. 2c). From the 263 CNV regions, 25 regions contained samples with duplications, 132 regions contained samples with deletions, and 24 regions contained samples with deletions as well as samples with duplications. Allele frequencies from the genotyped duplications and deletions in the PanC4 controls were consistent with percentages reported in the 1000 Genomes Project. We identified a median of 17 additional CNVs per sample by the mixture model that were not identified by the HMM (Figures S4 and S5). On average, CNVs spanned 6 SNPs (interquartile range (IQR): 5-8) and were 12.6kb in size (IQR: 10.9kb - 17.6kb).

For 85 of the regions with deletions, small log ratios consistent with homozygous deletions appeared in a subset of the identified batches. Multi-batch models fit to these data require heavy-tails to accommodate the extreme observations and the resulting mixture components potentially capture different latent copy number states between the batches. Rather than exclude these observations, CNPBayes augments the observed data with simulated deletions. For the small number of individuals with likely germline homozygous deletions, their posterior probabilities can be interpreted as having been influenced by an empirically derived prior. Posterior probabilities for the remaining mixture components tend to be nearly equivalent to a model without augmentation fit to a dataset excluding the rare observations. For example, the concordance of mixture component posterior probabilities comparing a model with augmentation to a model without augmentation that excluded 6 individuals with likely germline homozygous deletions at CNP_121 was near 1 (Figure S6).

**Comparison to other software**

Conceptually, our approach is most similar to CNVCALL [9] as Cardin *et al.* model one-dimensional summaries of CNV regions for each subject using a Bayesian hierarchical mixture of *t* distributions [9, 31]. Below, we compare CNVCALL and CNPBayes at deletion and duplication polymorphisms in the PanC4 study that cover a range of data quality issues encountered in practice. When necessary, we performed a stratified analysis on the low and high quality samples. As CNVCALL does not interpret the copy number of the mixture components identified, we have labeled the copy number of their assigned components using the approach described for CNPBayes. For CNVCALL, we have used the first principal component as a one-dimensional summary for the CNV regions as recommended. Finally, we compare these methods to a set of simulations derived from HapMap samples where the true copy number is known.

*PanC4 study*

We performed a detailed analysis of four CNV regions in the PanC4 study (CNP_121, CNP_128, CNP_100, and CNP_240) that capture a range of data quality and copy number states (Figures S6–S9). For CNP_121, CNPBayes identified 5 batches in the high quality samples and a single batch in the low quality samples (Figure S6). A three component model was selected and the components were mapped to copy numbers 0, 1, and 2 from the BAFs as previously described, generating copy number frequencies of 9, 422, and 7167 (Hardy Weinberg equilibrium (HWE) $\chi _{1}^{2}$=1.15, p=0.28). Of the 7598 samples, 17 individuals were not called by CNVCALL, including 8 individuals with a missing log _2_*R* ratio in the CNV region and the 9 zero-copy individuals identified by CNPBayes (HWE $\chi _{1}^{2}$ = 6.18, p = 0.01). For the remaining 7581 samples, the posterior probabilities were highly concordant for both approaches. Similarly, CNP_128 is a deletion polymorphism. No batch effects were detectable in either the low or high quality data collections by CNPBayes (Figure S7). CNVCALL discarded 181 individuals at this locus, including 4 homozygous deletions identified by CNPBayes. The observed counts for copy numbers 0, 1, and 2 from CNPBayes were 4, 317, and 7277 (HWE $\chi _{1}^{2}$ = 0.08, p=0.77), while the corresponding counts for CNVCALL were 0, 303, and 7,114 ($\chi _{1}^{2}$ = 3.23, p=0.07). For CNP_100, CNPBayes identifies 6 batches in both the low- and hiqh-quality samples (Figure S8) and detects a duplication in the high quality samples but not for the lower quality data. CNVCALL did not identify any copy number alterations whether fit to all samples or when restricted to the set of high quality samples. BAFs among samples with the duplication identified by CNPBayes were highly consistent with three copies. Finally, for CNP_240 CNPBayes identifies copy numbers 0-3 at frequencies 2, 228, 5757, and 39 ($\chi _{1}^{2}=0.03, p=0.87$ for copy numbers 0, 1, and 2) while CNVCALL identifies only hemizygous deletions (*n*=280) and diploid copy numbers (*n*=5647) ($\chi _{1}^{2}=3.47, p=0.06$) (Figure S9). Overall, these analyses indicate that for regions where the signal to noise ratio is high, CNPBayes generates posterior probabilities of the latent copy numbers that are highly concordant with CNVCALL. Substantial differences in the two approaches arise for rare CNVs and for CNVs where the mixture components have greater overlap, often attributable to batch-to-batch differences in technical variation that are more flexibly modeled by CNPBayes. The CNPBayes assignment of relatively rare, large negative log _2_*R* ratios to a copy number zero state was consistent with expected frequencies of a deletion allele segregating in the population.

*Simulation*

To benchmark the sensitivity and specificity of this approach when the true genotypes were known, we extracted high quality data from a subset of HapMap phase III samples (*n* = 990) processed on 16 chemistry plates and hybridized to Affymetrix 6.0 chips. A 109 kb region on chr 4 containing 1 SNP and 53 nonpolymorphic markers spans a deletion polymorphism with an allele frequency near 22%. We increased the level of difficulty for genotyping these samples by increasing the variance and/or shifting the location of the probe-level data in a subset of the chemistry plates. For each simulated dataset, we fit both CNVCALL and CNPBayes. While we did not provide the true batch labels to either method, CNPBayes estimated the batches from the plate surrogates. With no simulated batch effects, CNPBayes and CNVCALL had nearly identical performance with near perfect sensitivity and specificity (area under the receiver operator characteristic curve (AUC) > 0.99). However, for simulated datasets with batch effects in the mean or variance, accuracy of CNVCALL decreased by an average of 25% while performance characteristics of CNPBayes remained qualitatively similar (Figure S10).

**Risk model for pancreatic cancer**

**Fig. 4:**
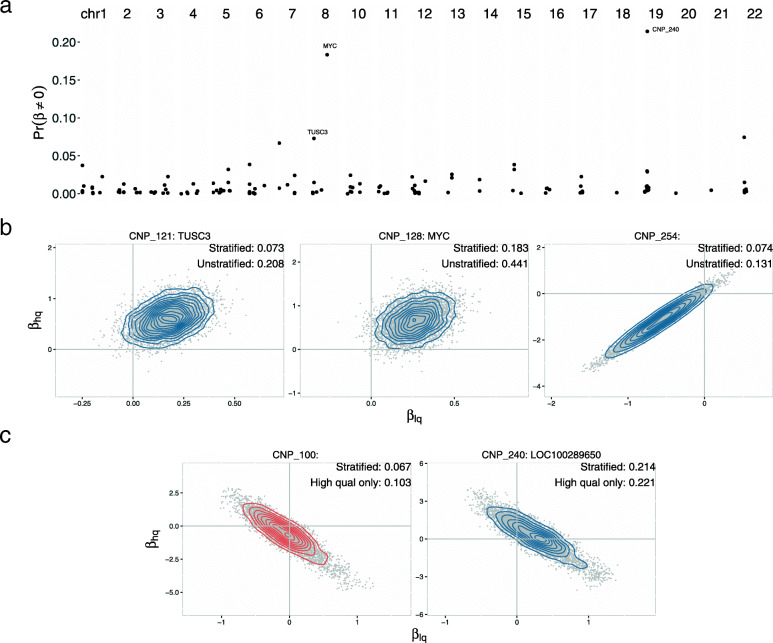
Bayesian regression models for pancreatic cancer risk. To incorporate uncertainty of the copy number assignment from the low-level data, the integer copy number was sampled from the subject-specific posterior probabilities provided by CNPBayes at each iteration of the MCMC. While batch effects on CNV inference were already accounted for in the low and high quality sample collections, an imbalance of the pancreatic cancer cases between these collections warranted a stratified model with an interaction between copy number and data quality and an indicator, *z*_*c*_, multiplying these coefficients that allowed the slopes to be exactly zero. **a** Posterior probabilities of association from the stratified model for CNV regions across the genome. For regions where copy number inference was unaffected by data quality and associated with pancreatic cancer risk, regression coefficients for the low and high quality collections were positively correlated and the posterior mean of *z*_*c*_ (upper right corner) increased in the more powerful unstratified analysis using all 7598 samples (**b**). By contrast, negatively correlated coefficients indicated an effect of data quality on CNV inference confirmed by visual inspection and the appropriate follow-up analysis and estimated probability of association was limited to the high quality sample collection (**c**)

To evaluate whether changes in germline copy number effect pancreatic cancer risk, we fit a Bayesian logistic regression model at each CNV region. Uncertainty of the copy number assignment for each participant was incorporated in the regression model by sampling the integer copy number from a multinomial distribution parameterized by posterior probabilities from CNPBayes at each scan of the MCMC. As case-control status was unevenly distributed between the high and low data quality sample collections ($\chi ^{2}_{1}$=13.1, p=0.0003), the regression model included an interaction between copy number and data quality (Methods) as well as a single binary parameter *z*_*c*_ multiplying both of these terms that allows the slopes to be exactly zero. The posterior mean of *z*_*c*_ provides an estimate of the probability of an association with copy number (Fig. 4 and Table S2). Additional covariates included age, gender, and the first three principal components previously estimated from the SNP genotypes [20].

Genome-wide posterior probabilities of association between copy number and pancreatic cancer risk were near zero for most CNV regions (Fig. 4a). For five CNV regions with non-zero probabilities, we assessed the joint distribution of the regression coefficients for the high and low quality samples (Fig. 4b and c). Participants with two copies of the Tumor Supressor Candidate 3, *TUSC3*, had a 20% increased odds of pancreatic cancer compared to individuals with germline hemizygous deletions in this gene (90% credible interval (CI) for odds ratio: 1.01 - 1.39). While the direction of this effect is inconsistent with its putative role as a tumor suppressor, up-regulation of *TUSC3* and possible oncogenic roles for this gene have been reported in cancers including non-small cell lung cancer, colorectal, thyroid, and head and neck cancers [32–35]. Among non-coding regions, we found that deletions for a CNV region in 8q24 were associated with a reduced risk of pancreatic cancer (90% CI: 1.09-1.59). Chromosome 8q24 has been implicated in many cancers and is known to contain regulatory elements for the tumor oncogene *MYC* located at 128,748,315-128,753,680 bp [36]. We have previously demonstrated the association of SNPs in this region with an increased risk of pancreatic cancer [37, 38]. As copy number regression coefficients at CNV regions spanning *TUSC3* and near *MYC* were positive and highly correlated for both the low and high quality sample collections, an unstratified analysis using all 7598 participants doubled the posterior probability of association for these genes (Fig. 4b). Overall, our approach provides conservative measures of the association between copy number and pancreatic cancer risk across the genome, accounting for latent batch effects and copy number uncertainty separately for samples where data quality was more compromised.

## Discussion

We performed a genome-wide analysis of germline copy number variants in the largest study to date of pancreatic cancer, implementing approaches to correct for latent batch effects and risk models that incorporate uncertainty of the copy number estimates. As the batch effects we identified were likely related to differences in PCR efficiencies that can vary across the genome and between groups of samples processed on different chemistry plates within a single laboratory (not between study sites), we identified and adjusted for batch effects in a region-dependent manner in contrast to alternative methods. Using this approach, nearly 70% of CNV regions analyzed had multiple batches that were related to chemistry plates and not the individual laboratories that contributed samples.

Using the methods outlined in this study, we found that germline deletions of *TUSC3* and near *MYC* were more prevalent among participants without pancreatic cancer. Germline deletions of these genes have not been previously implicated in pancreatic cancer, though upregulation of expression of these genes have been implicated in some cancers in an apparent tissue-dependent manner. Although this study did not evaluate whether deletions at these loci were well tagged by neighboring SNPs, phasing the nearby SNPs would allow direct inference for whether variation in copy number is associated with pancreatic cancer risk among participants with the same SNP haplotype [39, 40]. While we evaluated copy number at both known and HMM-discoverable CNV regions for pancreatic cancer risk, more sensitive technologies for identifying smaller CNV regions with potentially rare germline CNVs among cancer patients are needed, and will not be well tagged by neighboring SNPs. Whether mosaic copy number alterations in hematopoietic cells could further modulate risk has not been evaluated [41–44].

Finally, we assumed an additive model for integer copy number and the log odds of cancer risk. Dominant and recessive mechanisms of genotype-phenotype associations are possible and the evidence for these models using Bayes factors could be averaged with weights reflecting our a priori beliefs.

## Conclusions

Statistical inference predicated on measures of abundance such as DNA copy number are highly susceptible to batch effects, and the sources of these effects are not generally known. As studies become increasingly large-scale with inevitable batch effects and heterogeneity in sample quality, the scalable approach provided by CNPBayes will be helpful for modeling unwanted technical variation and avoiding the potential confounding between batch effects and copy number when evaluating disease risk.

## Supplementary information

**Additional file 1** Figure S1: Median absolute deviation and autocorrelation of autosomal log_2_*R* ratios. Figure S2: Preprocessing and quality control analyses. Figure S3: Frequency of CNV regions with 1 to 7 batches identified by grouping the eCDFs of the log_2_*R* summaries. Figure S4: Number of additional CNVs identified from the Bayesian mixture model. Figure S5: Technical variation within and between samples obscures identification of hemizygous deletions. Figure S6: A deletion polymorphism at CNP_121. Figure S7: A deletion polymorphism at CNP_128. Figure S8: A duplication polymorphism at CNP_100. Figure S9: A CNV region with both deletions and duplications evident in the high quality samples. Figure S10: Performance of CNV detection methods on HapMap data.

**Additional file 2** Supplemental Tables for Bayesian copy number detection and association in large-scale studies.

## Data Availability

The PanC4 study is available under dbGap accession number phs000206.v5.p3.

## Abbreviations

CNV: Copy number variant
HMM: Hidden Markov model
MCMC: Markov Chain Monte Carlo
PanC4: Pancreatic cancer case-control consortium
SNP: Single nucleotide polymorphism
ACF: Autocorrelation coefficient
eCDF: Empirical cummulative distribution function
K-S: Kolmogorov-Smirnov
MB: Multiple batches
SB: Single batch
MBP: Multiple batch pooled variance
SBP: Single batch pooled variance
HWE: Hardy Weinberg equilibrium
BAF: B allele frequency
PC: Principal component
IQR: Interquartile range
AUC: Area under the receiver operator characteristic curve
