# Supplementary material for: Bayesian copy number detection and association in large-scale studies
Source: BMC Cancer. 2020 Sep 7;20:856. doi: 10.1186/s12885-020-07304-3 (PMC7487704; doi:10.1186/s12885-020-07304-3)
Supplement: Supplementary file 1 — Additional file 1 Figure S1: Median absolute deviation and autocorrelation of autosomal log2R ratios. Figure S2: Preprocessing and quality control analyses. Figure S3: Frequency of CNV regions with 1 to 7 batches identified by grouping the eCDFs of the log2R summaries. Figure S4: Number of additional CNVs identified from the Bayesian mixture model. Figure S5: Technical variation within and between samples obscures identification of hemizygous deletions. Figure S6: A deletion polymorphism at CNP_121. Figure S7: A deletion polymorphism at CNP_128. Figure S8: A duplication polymorphism at CNP_100. Figure S9: A CNV region with both deletions and duplications evident in the high quality samples. Figure S10: Performance of CNV detection methods on HapMap data. [file 12885_2020_7304_MOESM1_ESM.pdf]

Bayesian copy number detection and association in large-scale studies

Stephen Cristiano[1,*], David McKean[2,*], Jacob Carey[1,*], Paige Bracci[3], Paul Brennan [4], Michael Chou[5], Mengmeng Du[6], Steven Gallinger[7], Michael G. Goggins[8,9], Manal Hassan [10], Rayjean Hung[7], Robert Kurtz[11], Donghui Li[12], Lingeng Lu [13], Rachel Neale[14], Sara Olson[6], Gloria Petersen[15], Kari Rabe[15], Jack Fu[1], Harvey Risch[13], Gary L. Rosner[1,10], Ingo Ruczinski[1], Alison P. Klein[2,5,9†], Robert B. Scharpf[1,2,†]

1 Figures

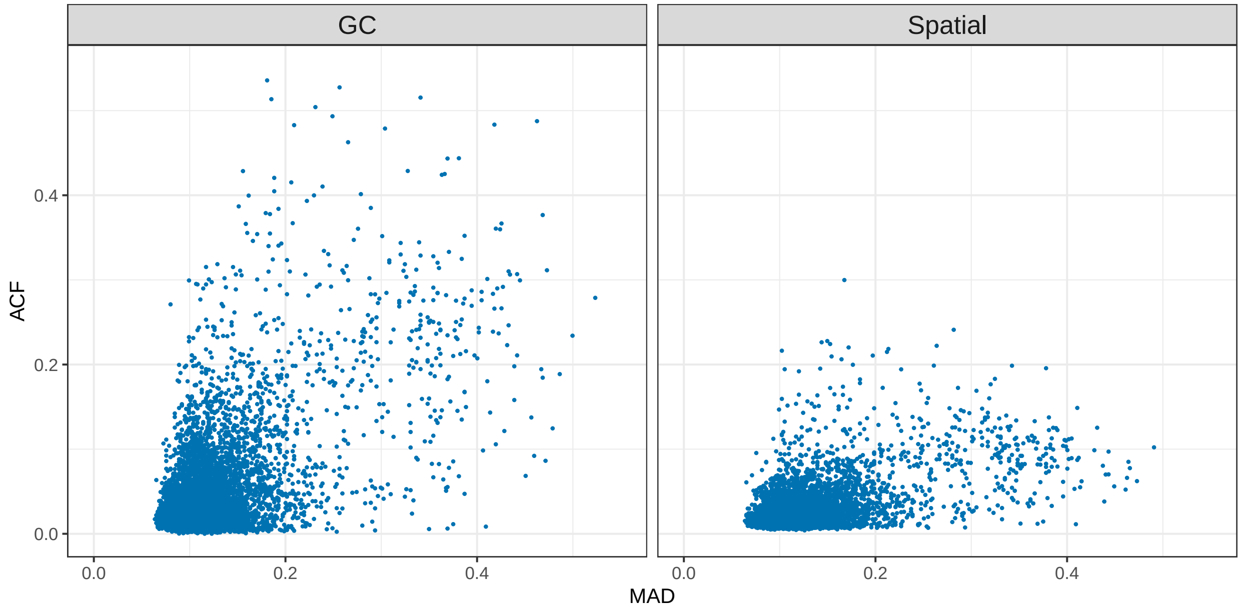

Figure S1: Median absolute deviation and autocorrelation of autosomal $\log_2 R$ ratios. After GC-correction, the majority of the samples have high data quality with median absolute deviations (MADs) less than 0.3 and lag-10 autocorrelations (ACFs) less than 0.1 (left). However, approximately 11% of the samples have autocorrelation greater than 0.1. To reduce autocorrelation, we applied a LOESS smoother to the scatterplot of genomic position versus $\log_2 R$ ratios using only SNPs that were likely heterozygous ($0.4 < \text{BAF} < 0.6$). From the LOESS model, we predicted the $\log_2 R$ ratios at all SNPs including those with BAFs outside the interval [0.4, 0.6]. The lag-10 autocorrelation of the residuals is less than 0.1 for nearly 98% of the PanC4 participants (right).

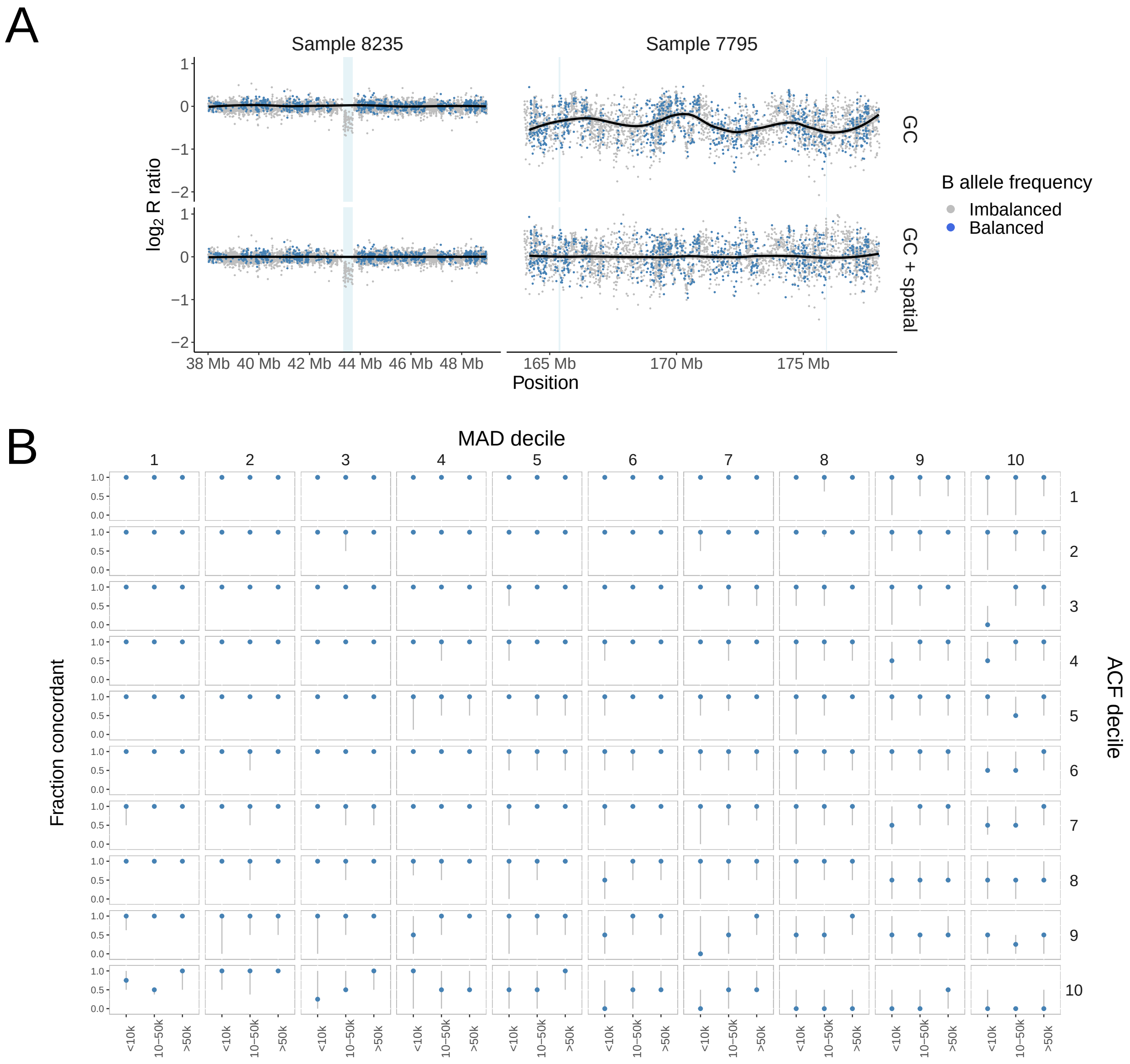

Figure S2: Preprocessing and quality control analyses. MAD and ACF measures of data quality among PanC4 participants were highly skewed to large values. **(A)** To reduce the ACFs, we smoothed the $\log_2 R$ ratios with balanced B allele frequencies by genomic position using loess and predicted the $\log_2 R$ ratios in regions with imbalanced B allele frequencies from the loess model. The predicted $\log_2 R$ ratios for SNPs in the hemizygous deletion in Sample 8235 (44 Mb) are all near zero (solid black line), resulting in negative residuals in the GC+spatial panel (bottom). Shading indicates hemizygous deletions that were identified from a HMM fit to the GC-only and GC+spatially corrected data. **(B)** We summarized the median concordance (blue) and interquartile range (gray) of the CNV calls from the HMM before and after the spatial loess model for all 7,598 samples in strata of MAD and ACF decile. The additional preprocessing has a neglible effect on CNV inference at the top left of this panel (low MAD and low ACF) and increases towards the bottom right (high MAD and high ACFs). We refer to the 1,560 samples in the highest decile of ACF (ACF $\geq$ 0.06) as samples of lower quality and the 6,038 samples in the first 9 deciles of ACF (ACF $<$ 0.06) as high quality samples.

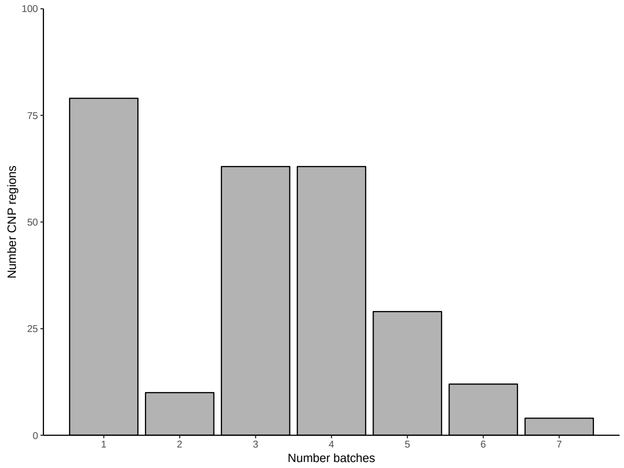

Figure S3: Frequency of CNV regions with 1 to 7 batches identified by grouping the eCDFs of the $\log_2 R$ summaries.

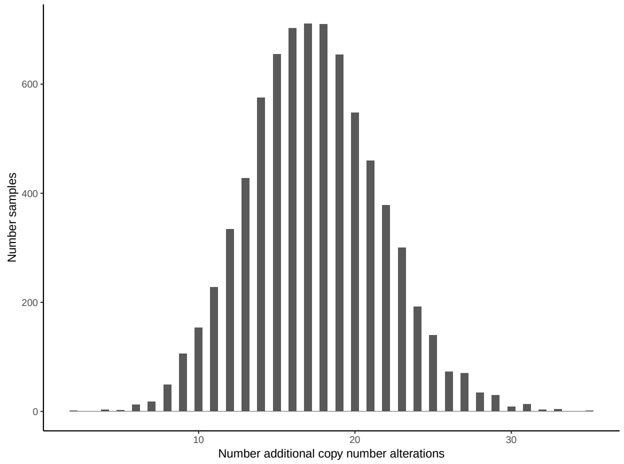

Figure S4: Number of additional CNVs identified from the Bayesian mixture model. On average, CNPBayes identified an additional 17 CNVs in each sample that were not detected from a hidden Markov model fit independently on the individual samples.

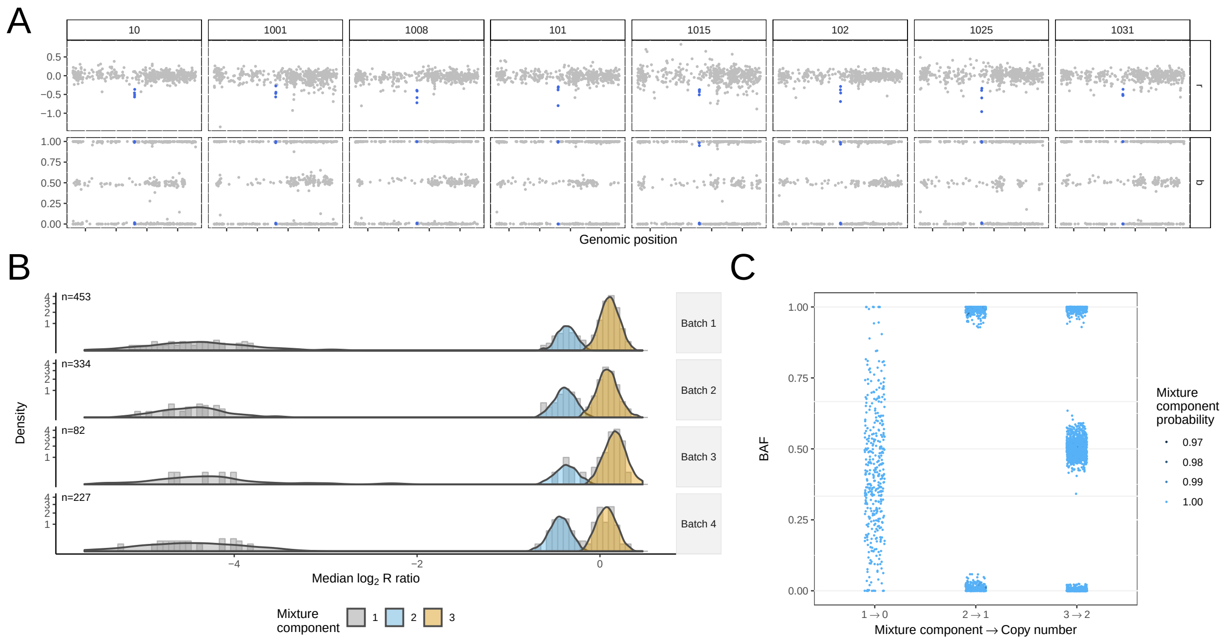

Figure S5: Technical variation within and between samples obscures identification of hemizygous deletions. (A) Log_2 R ratios (top) and B allele frequencies for SNPs in a 5kb CNP region (chr1:174,796,517-174,801,833) are highlighted in blue. The technical variation across the genome overwhelms the signal and the hemizygous deletions in these 10 samples are not detected. **(B)** The distribution of the median $\log_2 R$ for 6,038 samples. The black line overlaying the histogram of the observed data is the density of the posterior predictive distribution from the hierarchical Bayesian finite mixture model with mixture components color-coded by the mixture component indices. **(C)** B allele frequencies for SNPs spanned by the CNP region stratified by the inferred copy number state.

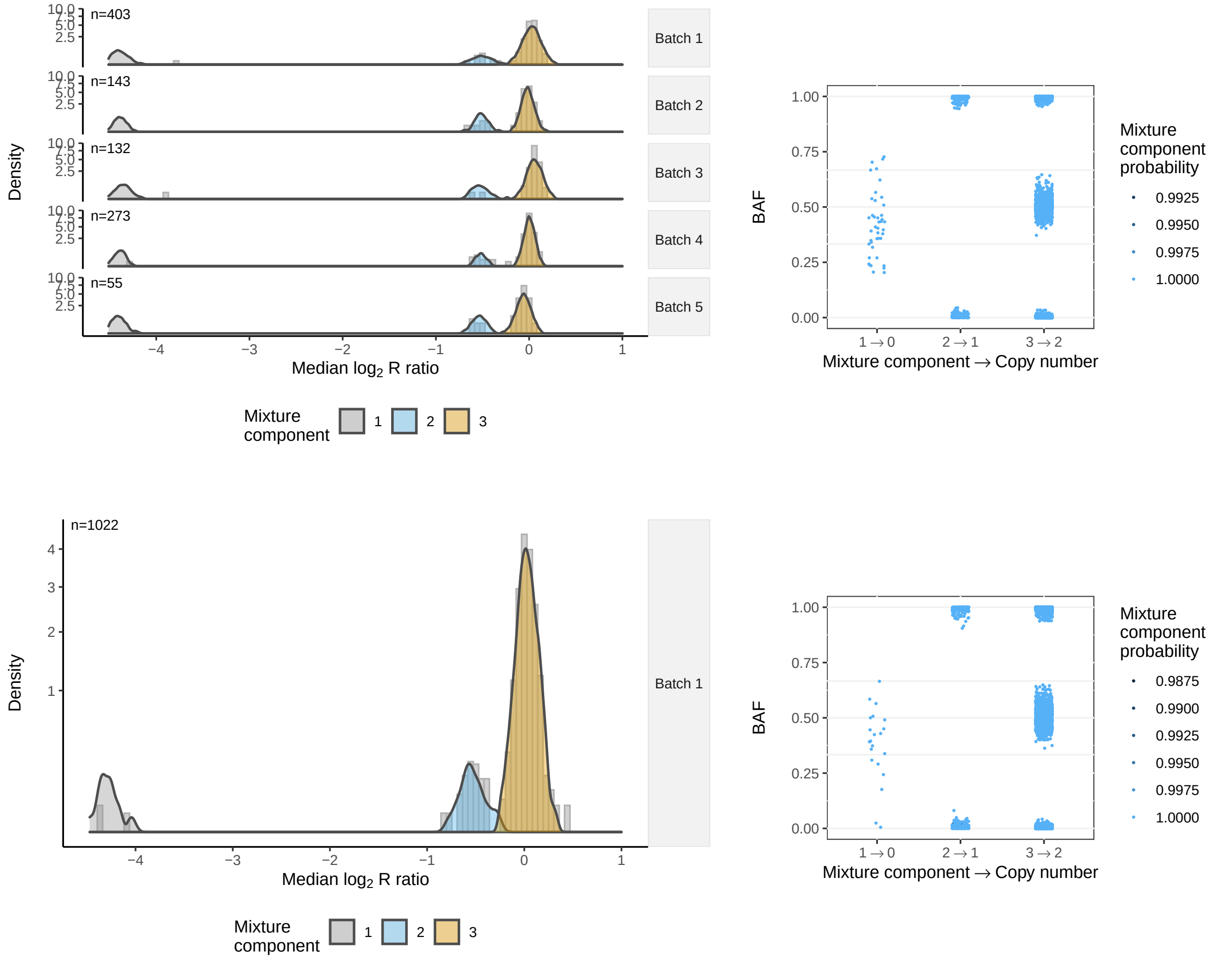

Figure S6: A deletion polymorphism at CNP_121. (A) Median $\log_2 R$ ratios for 6,038 high quality samples stratified into 5 batches (top) and 1,560 low quality samples (bottom). Solid density lines indicate the posterior predictive distribution from CNPBayes. **(B)** B allele frequencies are shaded by the posterior probability of the mixture component assignment and mapped to copy number states 0, 1, and 2. As expected for CNV regions with high signal to noise ratio, the mixture component labels were identical for components 1 and 2. Copy number frequencies for CNPBayes were 9, 422, and 7,167 for copy numbers 0, 1, and 2 (HWE χ^2=1.15, p=0.28). CNVCALL does not identify homozygous deletions that are rare at this CNP and excluded 8 samples with one or more missing values in the $\log_2 R$ ratios. Copy number frequencies of 0, 421, and 7,160 obtained from CNVCALL are not in HWE due to the absence of homozygous deletions (HWE χ^2 = 6.18, p = 0.01).

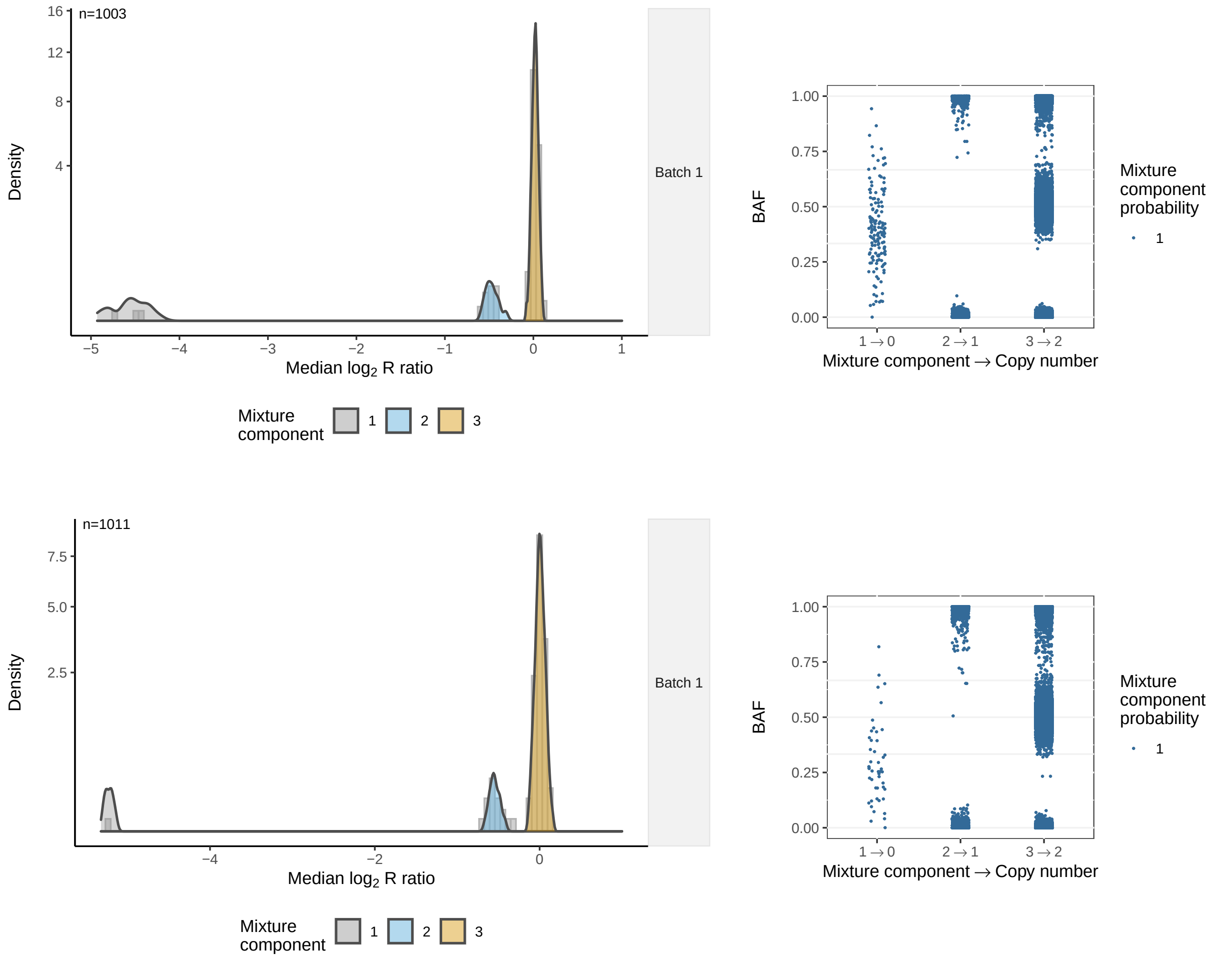

Figure S7: A deletion polymorphism at CNP_128. (A) Median $\log_2 R$ ratios for 6,038 high quality samples (top) and 1,560 low quality samples (bottom). No batch effects were detected by CNPBayes at this locus. **(B)** B allele frequencies shaded by the posterior probability of the mixture component assignment. Mixture component labels for CNVCALL and CNPBayes were identical for the second and third components, but the relatively rare homozygous deletions were not called by CNVCALL. The homozygous deletions identified by CNPBayes were consistent with HWE ($\chi^2 = 0.08$, p=0.77).

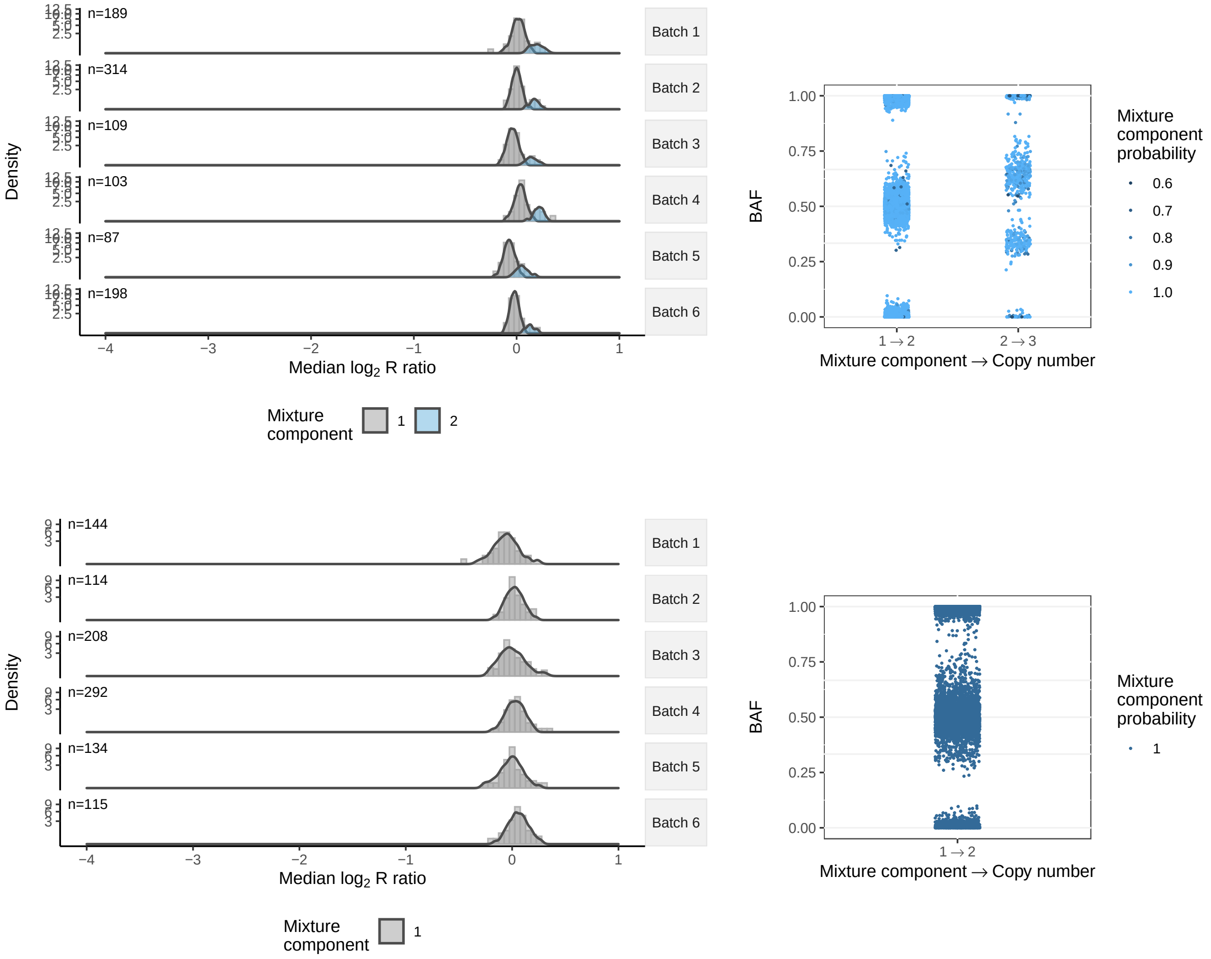

Figure S8: A duplication polymorphism at CNP_100. (A) CNPBayes identifies 357 individuals with a duplication in the high quality samples (top), but the higher noise in the low quality samples prevented their detection even with the multiple batches identified. CNVCALL did not identify any copy number changes at this locus. **(B)** B allele frequencies in the high quality samples (top) are consistent with a duplication event.

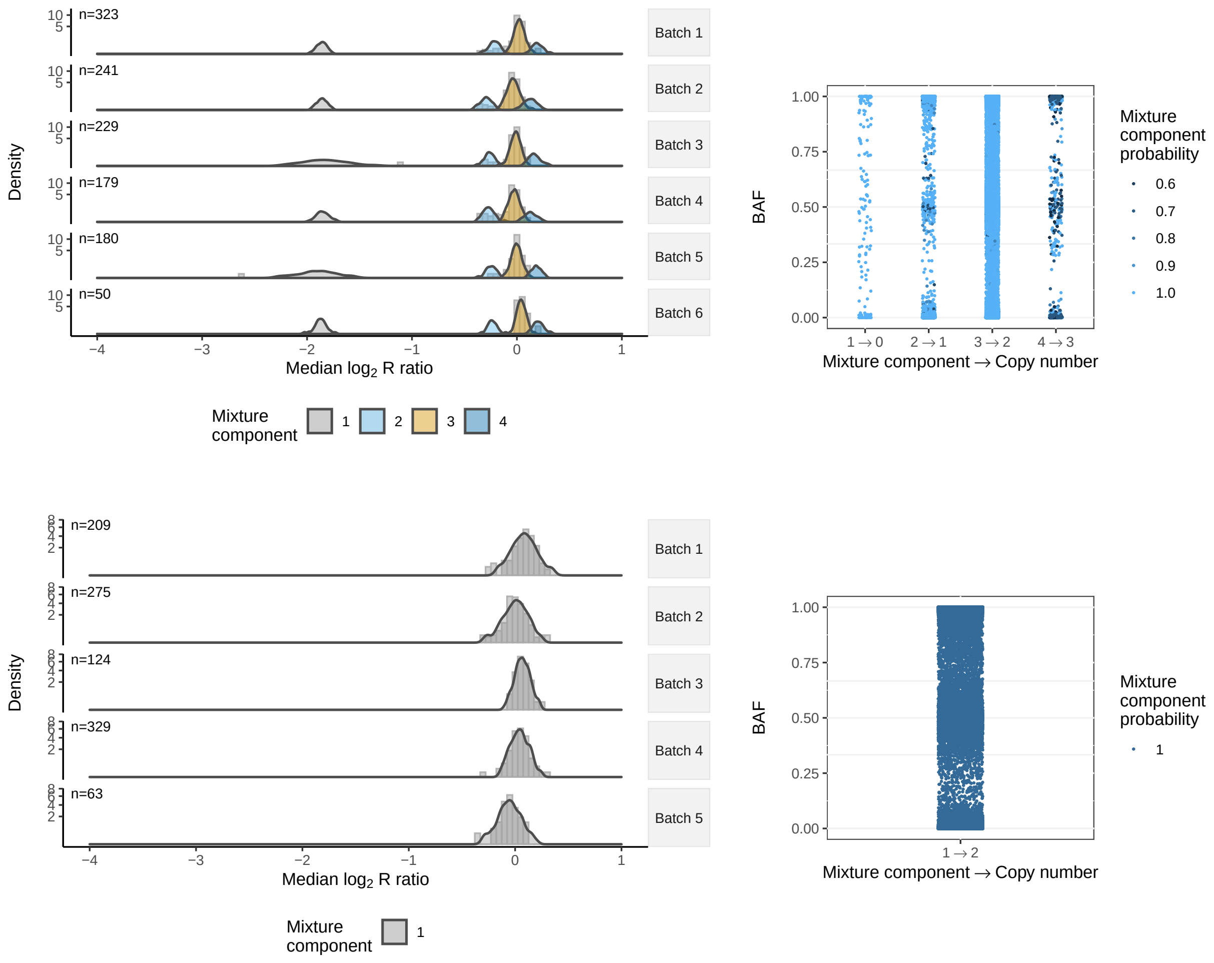

Figure S9: A CNV region with both deletions and duplications evident in the high quality samples. (A) CNPBayes employs a data augmentation step since the apparent homozygous deletions at CNP_240 appear in only 4 of the 6 batches. Many of the SNPs for samples with $\log_2 R$ ratios consistent with hemizygous deletions have BAFs that were inconsistent with copy number 1, likely indicating that the CNV region may be defined to broadly or that the region that is copy number altered may vary across individuals **(B, top)**. A one-dimensional summary obtained from a principal component analysis may be more powerful when the CNV boundaries differ, and is consistent with the identification of more hemizygous deletions by CNVCALL (280 versus 228). CNPBayes call frequencies were more consistent with HWE ($\chi^2 = 0.028$, p=0.087) than CNVCALL since rare homozygous deletions were not identified by CNVCALL ($\chi^2 = 3.47$, p=0.06).

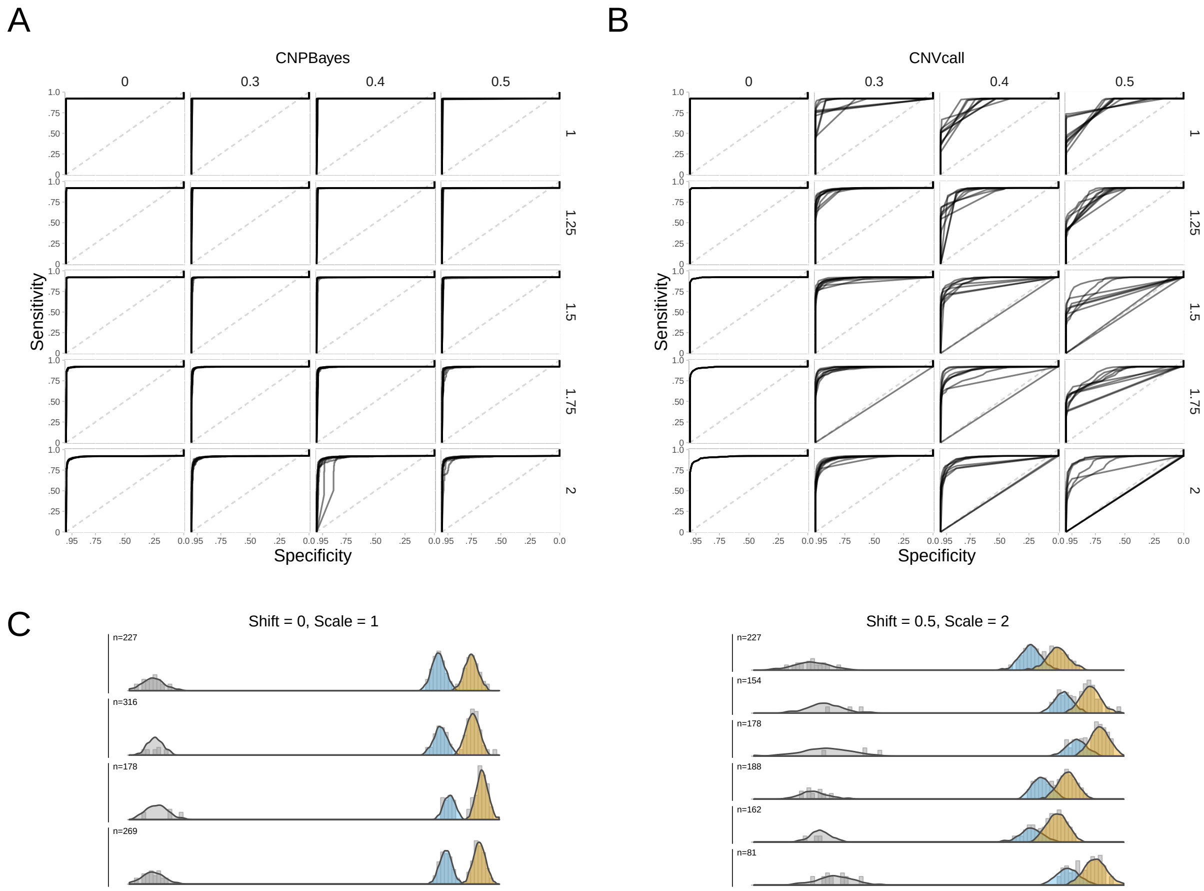

Figure S10: Performance of CNV detection methods on HapMap data. A deletion polymorphism for a 109 kb region on chromosome 4 in 990 HapMap samples that were processed on 16 chemistry plates. To simulate batch effects, a mean shift and/or rescaling of the variance was applied to the $\log_2 R$ ratios in a random subset of plates. **(A)** Each panel displays 10 receiver operator characteristic curves for CNPBayes evaluated on simulated datasets with the mean shift indicated in the column margins and scaling indicated in the row margins. While the true batch effect is not provided, CNPBayes attempts to infer the latent batches and has qualitatively similar performance as the level of difficulty increases from top-left to bottom-right. Two examples of the simulated data and posterior predictive distributions from CNPBayes are displayed in the top margin: no batch effect (left) and a mean shift of 0.5 with rescaling by factor of 2 (right). **(B)** By contrast, sensitivity and specificity of CNVCALL evaluated on the same data worsens as the simulated batch effects becomes more pronounced. **(C)** Representative simulations without (left) and with (right) batch effects.
